# Ontogenesis from embryo to juvenile and salinity tolerance of Japanese devil stinger *Inimicus japonicus* during early life stage

**DOI:** 10.1186/2193-1801-2-289

**Published:** 2013-07-01

**Authors:** Youji Wang, Lisha Li, Guoqiang Cui, Weiqun Lu

**Affiliations:** Key Laboratory of Exploration and Utilization of Aquatic Genetic Resources, Ministry of Education, College of Fisheries and Life Science, Shanghai Ocean University, 999 Huchenghuan Road, Shanghai, 201306 China

**Keywords:** Inimicus japonicus, Early development, Morphological characteristics, Larvae, Juvenile, Salinity tolerance

## Abstract

Embryonic development and morphological characteristics of Japanese devil stinger *Inimicus japonicus* during early life stage were investigated. Larvae were hatched out 50 h after fertilization at temperature 21°C. Total length of the newly hatched larva was 4.03 mm, the mouth of the larva opened at 3 days after hatching (DAH), and the yolk sac of the larva disappeared at 5 DAH. After hatching, the pectoral fin first developed, then the tail fin, dorsal fin, anal fin and pelvic fin continuously developed, and all fins formed completely at 15 DAH. The metamorphosis was complete at 25 DAH, and the body color and habit of the metamorphosed individuals were different from the larvae. At 30 DAH, the morphology and habit of the juveniles were the same to adults. In order to determine the suitable salinity for larviculture of *I. japonicus*, salinity tolerance at different early developmental stages was compared in terms of the survival activity index (SAI) and mean survival time (MST). The results indicated that salinity tolerance varied with development stages. The optimum salinity range for newly hatched larvae was 10–25‰. Larvae showed low tolerance to low salinity (5‰) before the mouth opened, and the suitable salinities for the larvae with open mouth, yolk-sac larvae, post yolk-sac larvae were 10–15‰. The flexion larvae showed a wider salinity tolerance with range of 5–20‰. After metamorphosis, the juveniles showed a preferable adaptability of salinities of 15–20‰. The SAI and MST of individuals at various stages under different salinity conditions were positively correlated.

## Introduction

The devil stinger *Inimicus japonicus*, a valuable demersal marine scorpaenid fish, is widely distributed along the coastal areas of eastern Asia with depth range 10-200m, where salinity fluctuates frequently due to rainfall in summer. During its reproductive season, the larvae may suffer environmental changes severely such as salinity fluctuation, but little is known about their salinity tolerance during their early stage. The devil stinger is one of the species for which artificial seed production and cultivation have been developed along the coastal areas of China and Japan to increase the harvest yield since the early 2000s, and it has been considered to be a new commercially important species to be introduced into the aquaculture industry (Takushima et al. 2003; Liu and Quan 2005; Kadomura et al. 2006; Chen et al. 2009; Kim et al. 2012). However, the wild population of the devil stinger has declined rapidly because of overfishing and habitat destruction, it is urgent to conduct relevant studies on resource conservation and artificial breeding. In China and Japan, some hatcheries have tried to establish seed production, but success has not yet been attained because of sudden mass mortality during the larviculture in recent years (Kim et al. 2012). Rearing conditions, egg quality, and diseases are suspected as causes for this (Kadomura et al. 2006). Information regarding its early life history and larviculture, which can provide useful information for developing conservation and management plans, has not been well reported. There is an urgent need for researchers to learn about the larval biology of this species, and to provide some useful information to culture this species.

There are some studies on reproductive biology and osteological development of *I. japonicus* (Imamura and Yabe 1997; Takushima et al. 2003; Nozaki et al.^2003^). The reproductive cycle of devil stinger has been investigated, and its spawning season is from May to August, with peaks from May to June (Nozaki et al. 2003). Although attempts have been made to establish seed production and entire aquaculture process for this species (Takushima et al. 2003; Liu and Quan 2005), the technique has not been fully developed, and studies on larval ecology are still lacking, especially the salinity tolerance during early life stage has not been elucidated. Sudden mass mortality during the larval rearing stage due to unknown causes is a serious problem. Inappropriate rearing or feeding conditions, defects in egg quality, and infectious diseases are suspected as causes of sudden mass mortality (Kim et al. 2012). It is therefore necessary to accumulate fundamental information on the larval biology of this species in order to establish the technique for artificial seed production.

Study on early life history characters of fish makes a fundamental key for enabling a closer approach to their biology and taxonomy (Meijide and Guerrero 2000; Celik et al. 2012). Morphological characteristics are very important as they provide information of life history of fish and critical reference to hatchery production (Martinez and Bolker 2003). In addition, studies on embryonic and larval development of any fish species can be useful in directing the husbandry efforts of fish breeder to the specific state and requirements of each development stage (Celik et al. 2012). Chen et al. (2009) investigated the feeding rhythm and lethal time during starvation of the devil stinger *I. japonicus*. However, detailed study about the embryonic and larval development of scorpionfish is scarce. In addition, information is lacking concerning ontogeny of Japanese devil stinger *I. japonicus* from egg to juvenile.

Salinity plays an important role in embryonic development, yolk sac absorption, larval and juvenile growth Boeuf and Payan (2001). Embryonic and larval stages are two sensitive periods during fish life history, changes in environmental conditions may cause negative effects on larval development, and inappropriate culture condition may result in mass mortality of larval fish. Thus it is useful to study the salinity tolerance of fish during early life stages and choose the suitable salinity for larviculture (Boeuf and Payan 2001). Some reports of the effects of salinity on growth and survival of larval fish, such as *Caranx mate* (Santerre 1976), brown-spotted grouper *Epinephelus tauvina* (Akatsu et al. 1983), gilthead sea bream *Sparus aurata* (Tandler et al. 1995) and Brazilian flounder *Paralichthys orbignyanus* (Sampaio et al. 2007) larvae, indicated an increase in survival and/or growth at intermediate salinities (>15 ppt but <30 ppt). Others found improved growth or survival of larvae at higher salinities (>34 ppt), such as milkfish *Chanos chanos* (Swanson 1996) and southern flounder *Paralichthys lethostigma* (Henne and Watanabe 2003; Moustakas et al. 2004). Moreover, no significant differences in growth were observed among different salinities in cobia *Rachycentron canadum* larvae (Faulk and Holt 2006). Thus, results vary among species and across developmental stages. The devil stinger *I. japonicus* is known to exhibit surface death from hatching to the first feeding stage during the process of larval production (Ruttanapornvareesakul et al. 2007), whether salinity can affect the survival in this period is still unclear. In the present study, the embryonic and larval development of laboratory-reared *I. japonicus* from egg to juvenile were described in detail, major morphological changes during larval development were investigated. In addition, salinity tolerance of devil stinger during early life stage was investigated. Survival activity index (SAI) and mean survival time (MST) of larvae, which are expressed as functions of tolerance to starvation of larvae (Furuita et al. 2000; Matsuo et al. 2006), have been used as effective indexes for assessment of salinity tolerance in larval *I. japonicus*.

## Materials and methods

### Broodstock maintenance

Thirty males (body weight 600g ) and thirty females (body weight 300g ) of *I. japonicus* were purchased from Ningde Fish market (Ningde, Fujian province), and were used as broodstock in the experiment. They were fed with commercial seawater fish feeds (Guangdong Yuehai Feed Group, Guangdong, China; Protein: 39%, Fat: 5%, Fibre: 3%, Ash: 15%, Moisture: 10%), three times a day. During broodstock culture, water temperature, pH, salinity and DO were monitored daily at 21 ± 0.5 °C, 8.0–8.1, 28–30‰ and 7.0–8.0 mg l^-1^ respectively. The photoperiod was maintained at 12L/12D by fluorescent lighting (lights on: 07:00–19:00 hours). Broodstocks were kept in two 500-l tanks. Female spawning was induced by intraperitoneally injecting luteinizing hormone-releasing hormone analogue (LRHA3) and human chorionic gonadotrophin (HCG). The doses of these two hormones for female were 5 μg/kg and 800 IU/kg, and males were injected with half doses of them. Spawning was observed 50 h after injection.

### Observations and measurements of embryos and larvae

Fertilized eggs were collected and incubated in 500-l tanks filled with clean seawater (30‰). The incubation tank was held at temperature 21.0°C and dissolved oxygen 7.0–8.0 mg l^-1^. Some of them were transferred into a beaker (500 ml) for embryonic development observations. Eggs were observed from spawning to hatching under an electron microscope (OPTON EM10C, Carl Zelss Company, Germany, No.5166, voltage is 60KV) and photographed using a colour video camera (Panasonic ZS10, Japan). Embryonic development stages were identified according to Jones et al. (1978) and Kimmel et al. (1995).

**Figure 1:**
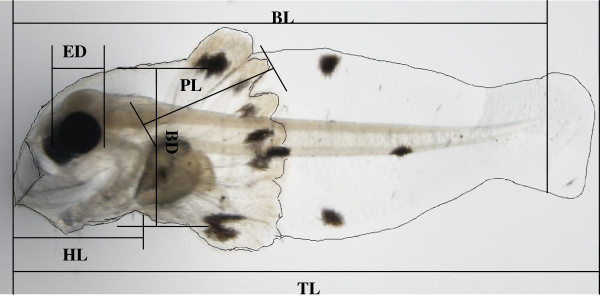
**Morphometric characters measured in the devil stinger*Inimicus japonicus*larvae, body depth (BD), eye diameter (ED), head length (HL), pectoral fin length (PL), body length (BL) and total length (TL).**

Newly hatched larvae were reared in incubation tanks and the density was maintained at 2×10^4^ ind. m^-3^ from 1 day after hatching (DAH) to 5 DAH. From 6 DAH to 11DAH, the water was changed 30% everyday and the density was reduced to 1×10^4^ ind. m^-3^. From 12 DAH to 25DAH, the water was changed 50% daily, and the culture density was 5×10^3^ ind. m^-3^. After 26 DAH, the fish were transferred to tanks which were circulated by flowing seawater, and the culture density was 1000 ind. m^-3^. The larvae were fed with rotifers and chlorella from 3 DAH to 20 DAH; and *Artemia nauplii* from 12 DAH to 30 DAH. From 20 DAH, artificial diets were supplied to the fish until the end of the experiment. Larvae were randomly sampled (n = 10) daily from hatch to 50 DAH. These specimens were observed under a dissecting microscope (JAPAN ASONE, IS/Mill-E, China) equipped with TSView software. On the other hand, samples were used for observations on general morphology and for the following morphometric measurements (Figure 1): body depth (BD), eye diameter (ED), head length (HL), pectoral fin length (PL), body length (BL) and total length (TL). Larval developmental stages were identified according to Kendall et al. (1984) and differentiated into six periods, I: newly hatched larva (1 DAH), II: yolk-sac larva (2 DAH), III: mouth-open larva (3 DAH), IV: post yolk-sac larva (5 DAH), V: flexion larva (15 DAH) and VI: juvenile (25 DAH).

### Salinity tolerance test at different developmental stages

Salinity tolerance test was conducted following the method of Matsuo et al. (2006). Selected developmental stages included: newly hatched larva (1 DAH), yolk-sac larva (2 DAH), mouth-open larva (3 DAH), post yolk-sac larva (5 DAH), flexion larva (with complete pectoral fin, 15 DAH) and juvenile (metamorphosis completed, 25 DAH). Ten salinities were selected for testing the salinity tolerance of *I. japonicus* for the five larval stages, including 5, 10, 15, 20, 25, 30, 35, 40, 45 and 50‰, and five salinities (15–35‰) were set for juvenile. Seawater with different salinities was made by adding red sea salt (Red Sea) into freshwater. Salinity was determined using a handheld refractometer and a multiparameter water quality meter (YSI Professional Plus). At each specific developmental stage, 900 larvae (450 juveniles) were sampled from the rearing tanks, and allocated into thirty plastic containers containing 950ml water with ten salinities (three containers for each salinity). The larvae were kept in static water without feeding, and other environmental conditions were the same to the above mentioned. Dead larvae were counted and removed with 300 ml of seawater by glass pipette, and 300 ml of fresh seawater was added once daily. Cessation of opercular movements and failure to respond to gentle prodding were the criteria used for death. This procedure was repeated until all fish died. The indexes for salinity tolerance used for the studies were survival activity index (SAI) and mean survival time (MST). SAI is expressed as a function of tolerance to starvation of larvae, positively correlated to the survival of larvae, and therefore defined as an index for larval quality of scorpionfish species (Matsuo et al. 2006). Mean survival time (MST) is defined as the mean survival time for all individuals in an experimental group over a 10-day period following direct transfer from salinity of pre-exposure to different salinities in this study (Watanabe et al. 1985). From the number of surviving larvae and survival duration (days), the SAI was calculated from the following equation:

where *N* is the total number of supplied larvae, *hi* is the cumulative mortality by the day *i*, and *k* is the number of days elapsed until all larvae died due to starvation. The average SAI was calculated for each batch and was used for further analysis.

### Statistical analysis

Data on salinity tolerance at six stages were statistically analysed using one-way analysis of variance (ANOVA), differences were considered significant at *P <* 0.05, and Student-Newman-Keuls post hoc multiple range tests were carried out to determine which treatments were different. Prior to the analysis, normality of the data was evaluated by using the Shapiro-Wilk’s W test and homogeneity of variances was checked by Levene’s test using the statistical software SPSS 17.0. The results are expressed as the means ± S.D. of the data.

## Results

### Embryonic development

**Figure 2:**
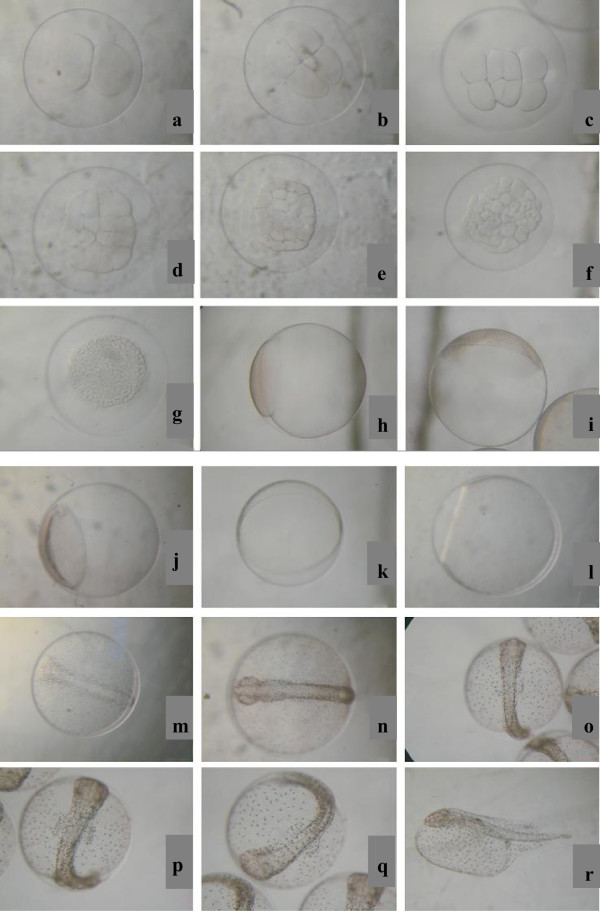
**Embryonic development of*Inimicus japonicas,*a:2-cell stage; b: 4-cell stage; c: 8-cell stage; d: 16-cell stage; e: 32-cell stage; f: 64-cell stage; g: Morula stage; h: Early blastula stage; i: Late blastula stage; j: Early gastrula stage; k: Mid gastrula stage; l: Late gastrula stage; m: Embryoid body formation; n: Formation of optic vesicle; o: Appearance of myomere; p: Efficiency stage of muscles; q: Pre-hatching stage; r: Newly hatched larva.**

The egg was buoyant, transparent and spherical in shape, lacking oil droplet. The mean diameter of the egg was 1.40 ± 0.05 mm. The cleavage of eggs was meroblastic and the first cleavage (two-celled stage) occurred within 0:23 hours after spawning (Figure 2a). Blastodisc divided to form two equal cells. The second cleavage occurred 0:32 hours and four blastomeres were clearly observed (Figure 2b). Blastodisc divided via meridional cleavage to form four equal cells. Then the eggs cleaved into 8 and 16 cells respectively. The third cleavage was horizontal and resulted in a 2× 4 array (Figure 2c). The forth cleavage occurred in two separate planes, cleavage furrow parallel to second cleavage plane and resulted in a 4× 4 array (Figure 2d). Eight and 16 cell stages were observed at 0:40 hours and 1:02 hours respectively (Figure 2c and d). The fifth cleavage took place after 1:35 hours from spawning (Figure 2e). Blastoderm divided via meridional cleavage into 32 cells and the 32 blastomeres were formed. After the sixth cleavage with 64 cells at 2:05 hours, the cells became smaller and were arranged irregularly (Figure 2f). At 4:05 hours, all blastomeres congregated like a mulberry, the animal pole uplifted like a hillock, and cell sizes varied differently (Figure 2g). The early blastula stage occurred at the vegetal pole 6:30 hours after spawning (Figure 2h). At this stage, the crowded cells expanded over the yolk and the blastomeres were divided asynchronously. The late blastula stage consisted of a multicellular blastomere (Figure 2i) and fully completed at approximately 9:40 hours. The gastrulation started at 11:36 hours after spawning (Figure 2j). Blastoderm cells spread over the yolk and epibolic cells increased at this stage. The embryo reached 50% epiboly at 13:45 hours after spawning and the blastoderm covered 50% of the yolk (Figure 2k). 75% epiboly stage was completed at 15:00 (Figure 2l). Neurula appeared at 19:47 hours, the prototype of the neural plate formed, head part uplifted, yolk plug exposed, pigments on the embryonic shield and yolk sac can be seen (Figure 2m). Pharyngula stage began at 22:23 hours, and a pair of kidney-shaped optic vesicles on both sides of the head was observed at this point (Figure 2n). At 25:46 hours, the embryoid surrounded the yolk sac, in the center of the embryoid, 8–11 myomeres formed (Figure 2o). The formation of the otic capsule started at 38:35 hours and embryo began to spin at this time (Figure 2p). The eye development and heart beat took place and body movement in the capsule was observed at 42:45 hours (Figure 2q). Larvae were hatched out at 44:05 hours, firstly, the head came out of the capsule, and then the tail swung hard to get off the capsule (Figure 2r). Hatching rates were 85–90% in aquarium at 50 h after spawning. The complete embryonic development was summarized in Table 1.

**Table 1:** **Embryonic development stages of*Inimicus japonicus*at 21°C**

Main stages	Substages	Time (h:min)	Description	Figure
Zygote	2-cell stage	0:23	First cleavage, blastodisc divided via meridional cleavage to form two equal cells	1a
	4-cell stage	0:32	Second cleavage, dividing the blastodisc into 4 blastomeres	1b
	8-cell stage	0:40	Third cleavage, 2 x 4 array of blastomeres	1c
	16-cell stage	1:02	Fourth cleavage, 16 blastomeres can be seen	1d
	32-cell stage	1:35	Fifth cleavage, 2 regular tiers (horizontal rows) of blastomeres, sometimes in 4 x 8 array	1e
	64-cell stage	2:05	Sixth cleavage, 64 blastomeres were ranked irregularly	1f
	Morula stage	4:05	The blastomeres were still distinct but the number of blastomeres can not be counted	1g
Blastula	Early blastula stage	6:30	The blastomeres were no longer distinguishable, the blastocoel began to form, and endoderm germ layer appeared	1h
	Late blastula stage	9:40	Epibolic cells increased, the archenteron can be seen, endoderm germ layer invaginated and the ectoderm layer formed	1i
Gastrula	Early gastrula stage	11:36	Blastoderm cells begin to spread over the yolk, and blastoderm remains uniform in thickness	1j
	Mid gastrula stage	13:45	Germ ring epiboled 1/2 of yolk sac, embryonic shield visible from animal pole	1k
	Late gastrula stage	15:00	75% coverage of the yolk cell by the blastoderm, dorsal side distinctly thicker; epiblast, hypoblast, evacuation zone visible	1l
Neurula	embryoid body formation	19:47	The prototype of the neural plate appeared, head part uplifted, yolk plug exposed, pigments on the embryonic shield and yolk sac can be seen	1m
Pharyngula	Formation of optic vesicle	22:23	On both sides of the head, a pair of kidney-shaped protrusions can be seen	1n
	Appearance of myomere	25:46	Embryoid surrounded the yolk sac, in the center of the embryoid, 8–11 myomeres can be seen.	1o
	Muscular effect	38:35	Embryo begins to spin frequently, heart beat 70-75/min	1p
	Pre-hatching stage	42:45	The embryo shows conspicuous muscular contractions	1q
Hatching	newly hatched larva	44:05	General transparent, floating on the water surface	1r

### Larval development and morphological observations

**Figure 3:**
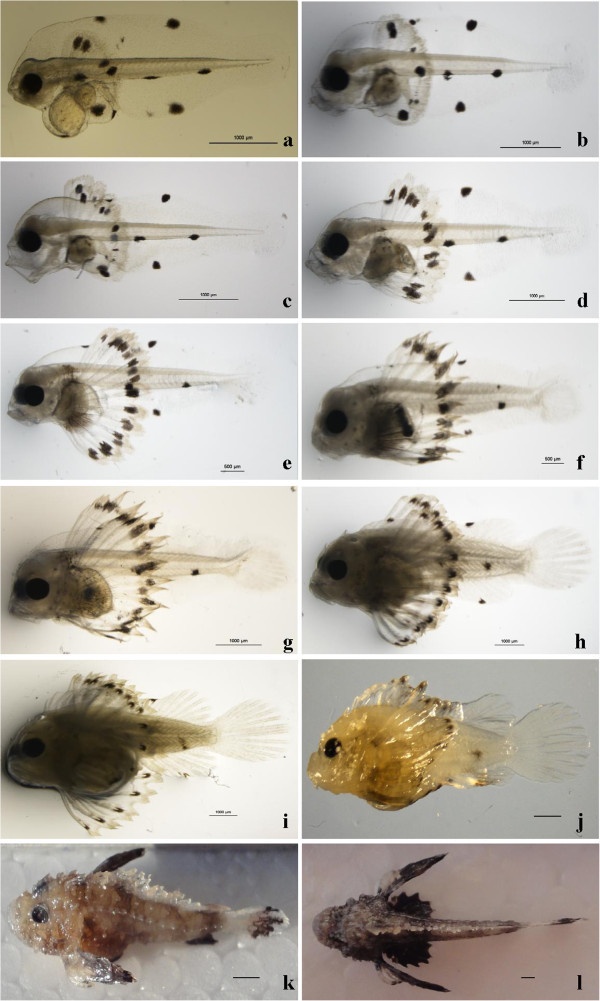
**Larval development of*Inimicus japonicas*, a: Post-hatching stage, 1 DAH; b: yolk-sac stage, 2 DAH; c: Larvae with mouth opened, 3 DAH; d: Post yolk-sac stage, 5 DAH; e: Preflexion larva, exogenous feeding, 8 DAH; f: Preflexion larva, 10 DAH; g: Flexion stage, notochord flexion started, 13 DAH; h: Postflexion larva, swim bladder with two chambers was visible 15 DAH; i: Postflexion larva, 20 DAH; j: End of metamorphosis, 25 DAH; k: Juvenile of 30 DAH; l: Juvenile of 40 DAH.** Scale bars = 1 mm.

Newly hatched larvae (TL: 4.03 ± 0.15 mm) in the post-hatching stage were laterally compressed and initially elongated. The head was closed to the yolk sac and the yolk sac was more than 50% of the total length, and the eyes were still unpigmented (Figure 2r). The body was transparent but pigmentation e.g., melanin and yellow pigments have appeared in the whole body (Figure 2r). 1DAH (TL: 4.23 ± 0.19 mm, Figure 3a), the yolk sac was reduced like a ball. The mouth and anus were closed and the undifferentiated alimentary tract appeared as a short tube. Eyes were not pigmented and three big spots of melanophores were scattered on the edge of body. The primordial pectoral fin fold was well developed in the sagittal plane but no fins were differentiated. A bigger round black spot were observed on the base of pectoral fin. 2 DAH (TL: 4.38 ± 0.11 mm, Figure 3b), the yolk sac became smaller, pigmentation increased over the eyes and the body but they were still translucent. Black melanophores were scattered on the head region, ventral and dorsal side of the body. The digestive track was a little inflated and dark. The primordial fin was slightly differentiated, no anal and dorsal fins were differentiated but pectoral fin bud was present. The larvae could not swim actively but short periods of swimming were observed. 3 DAH (TL: 4.54 ± 0.16 mm, Figure 3c), the eyes were pigmented, the mouth and anus opened, and the larvae started to feed exogenously. The pectoral fin was obvious with some big pigment spots. Swimming activity increased and the pectoral fin spread like a fan to maintain balance. 5 DAH (TL: 4.89 ± 0.21 mm, Figure 3d), the yolk sac has been completely absorbed and the larvae started to swim actively. The pectoral fin increased beyond the body depth. The eyes became very prominent and were fully pigmented. The larvae displayed phototaxis. There was a big black spot at the end of each ray of the spokewise pectoral fin. Pigmentation increased on the head and lateral parts of the body, black pigments were dominant, but yellow pigments were also present. The larvae swam very well. 8 DAH (TL: 5.17 ± 0.23 mm, Figure 3e), the pectoral fin increased, and the edge was wavy, yellow pigments were dense at the edge of the fins. The digestive tract was full of food. At this point, the larvae were pelagic and swam using the pectoral fin. 10 DAH (TL: 5.48 ± 0.32 mm, Figure 3f), pectoral fins were well developed with 9 rays, dorsal and anal fins began early differentiation. The caudal-fin rays formed. The notochord end was slightly flexed. There were clusters of pigment over the body. The larvae swam very well. 13 DAH (TL: 5.85 ± 0.29 mm, Figure 3g), the number of caudal-fin rays increased, but the dorsal and anal fins showed no difference compared with 10 DAH larvae. There were clusters of yellow pigment on the pectoral fin base. 15 DAH (TL: 5.92 ± 0.33 mm, Figure 3h), anal and dorsal fins began to develop and caudal fin rays were developed. The stomach of larvae contained food, ventral region of larvae was swollen and orange. Spinous protuberances were present on the head and opercular. 20 DAH (TL: 10.76 ± 0.63 mm, Figure 3i), the body color was pale yellow, and dorsal, caudal and anal fins differentiated well. The black spots on the dorsal and anal fins disappeared. A gold yellow zone on the pectoral fin was observed. The fish changed swimming to settling on the bottom of the tank. 25 DAH (TL: 12.06 ± 0.54 mm, Figure 3j), morphological metamorphosis was completed and the larvae completely transformed into juveniles. 30 DAH (TL: 15.65 ± 0.93 mm, Figure 3k), the morphology of the fish was similar to the 25 DAH fish, but the pigmentation increased significantly. The body shape and pigmentation pattern were similar to the adult fish. Yellow and black stripes were present on the body and fins. 40 DAH (TL: 19.10 ± 1.22 mm, Figure 3l), the body was almost completely covered with pigment. All fins developed well. The color of the body was tawny, red and yellow spots spread on the fins.

**Figure 4:**
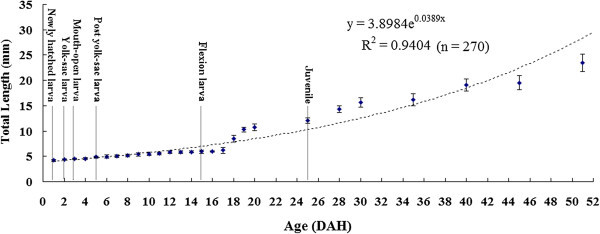
**Growth of*Inimicus japonicas*larvae from hatch to 51 DAH.** Each point represents the mean total length ± SD.

Growth of the black skirt tetra larvae followed an exponential curve during the larval stages and is represented by the equation y = 3.8984e^0.0389x^ (R^2^ = 0.9404, n = 270 where y is total length (TL) mm and x is DAH (Figure 4). Six larval development stages were observed after hatching; newly hatched larva, yolk-sac larva, mouth-open larva, post yolk-sac larva, flexion larva, and juvenile. The yolk sac has been completely consumed at 5 DAH. Notochord has been flexed between 13 DAH and 15 DAH. All the meristic characters were completely developed and juvenile stage started at 25 DAH.

### Salinity tolerance of larvae at different developmental stages

**Figure 5:**
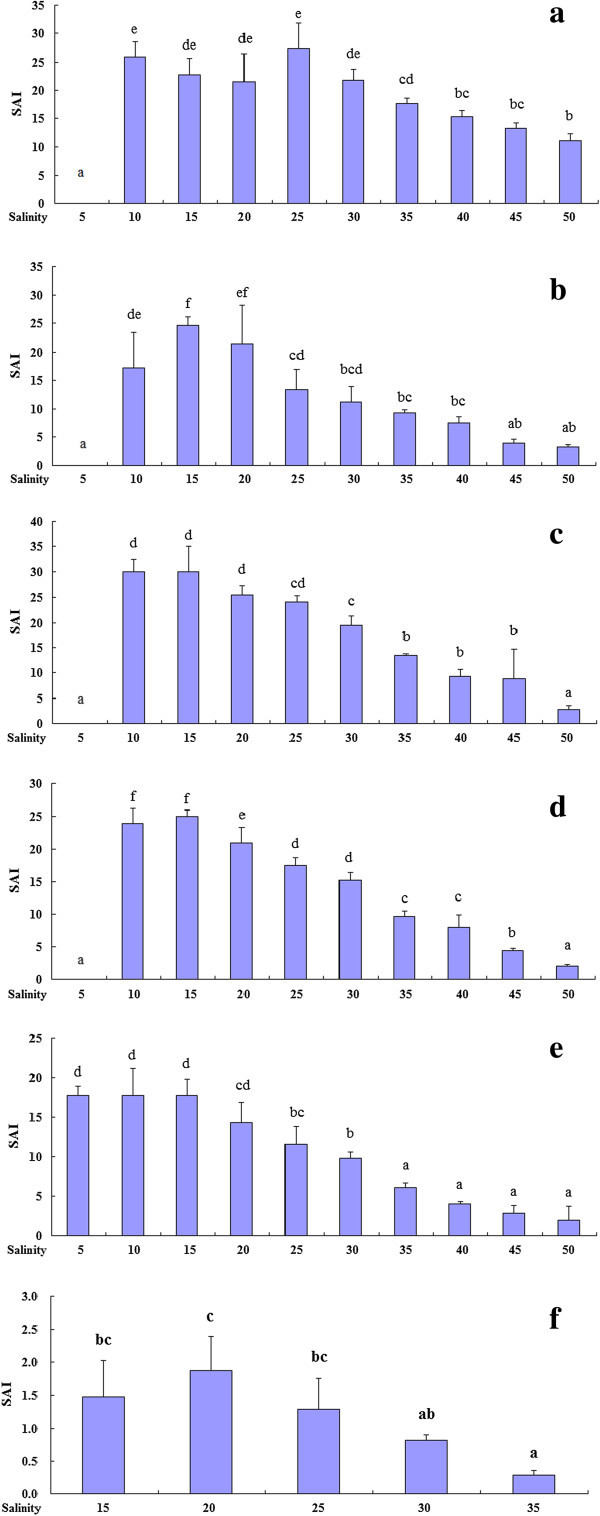
**Survival activity index under different salinities at different developmental stages in the*Inimicus japonicas,*a: newly hatched larva (1 DAH), b: yolk-sac larva (2 DAH), c: mouth-open larva (3 DAH), d: post yolk-sac larva (5 DAH), e: flexion larva (15 DAH) and f: juvenile (25 DAH).**

One-way ANOVA results on the effects of salinities on SAI in different developmental stages were summarized in Table 2. For the newly hatched larvae, the value of SAI was zero at salinity 5 and high values with no significant difference were present among salinity 10–30‰, and then decreased with salinity (Figure 5a). For the yolk-sac larvae, the highest SAI was observed at salinity 15‰, then the SAI decreased with salinity increase, and SAI values under salinity 15–20‰ were significantly higher than that at other salinities (Figure 5b). The similar trends were also observed for mouth-open larva and post yolk-sac larvae (Figure 5c & d), with both higher SAI values under medium salinities. However, the flexion larvae showed a wide salinity tolerance with a higher SAI value under salinity 5–20‰ (Figure 5e). For the juvenile, the highest SAI was observed at salinity 20‰ among five salinities, but showed no significant difference between salinity 15‰ to 20‰ (Figure 5f).

**Table 2:** **Summary of one-way ANOVA results on the effect of salinity on Survival activity index (SAI) and Mean Survival Time (MST) of*Inimicus japonicus*at early stage**

Parameter	Stage	df	MS	F	P-value
SAI	Newly hatched larva	9	199.589	29.744	<0.001
	Yolk-sac larva	9	194.737	18.001	<0.001
	Mouth-open larva	9	363.965	47.469	<0.001
	Post yolk-sac larva	9	249.507	130.224	<0.001
	Flexion larva	9	122.846	34.973	<0.001
	Juvenile	4	1.128	6.876	0.006
MST	Newly hatched larva	9	13.498	277.921	<0.001
	Yolk-sac larva	9	13.003	63.454	<0.001
	Mouth-open larva	9	21.216	69.987	<0.001
	Post yolk-sac larva	9	16.872	280.507	<0.001
	Flexion larva	9	6.357	30.312	<0.001
	Juvenile	4	0.627	6.56	0.007

Figure legends.

**Figure 6:**
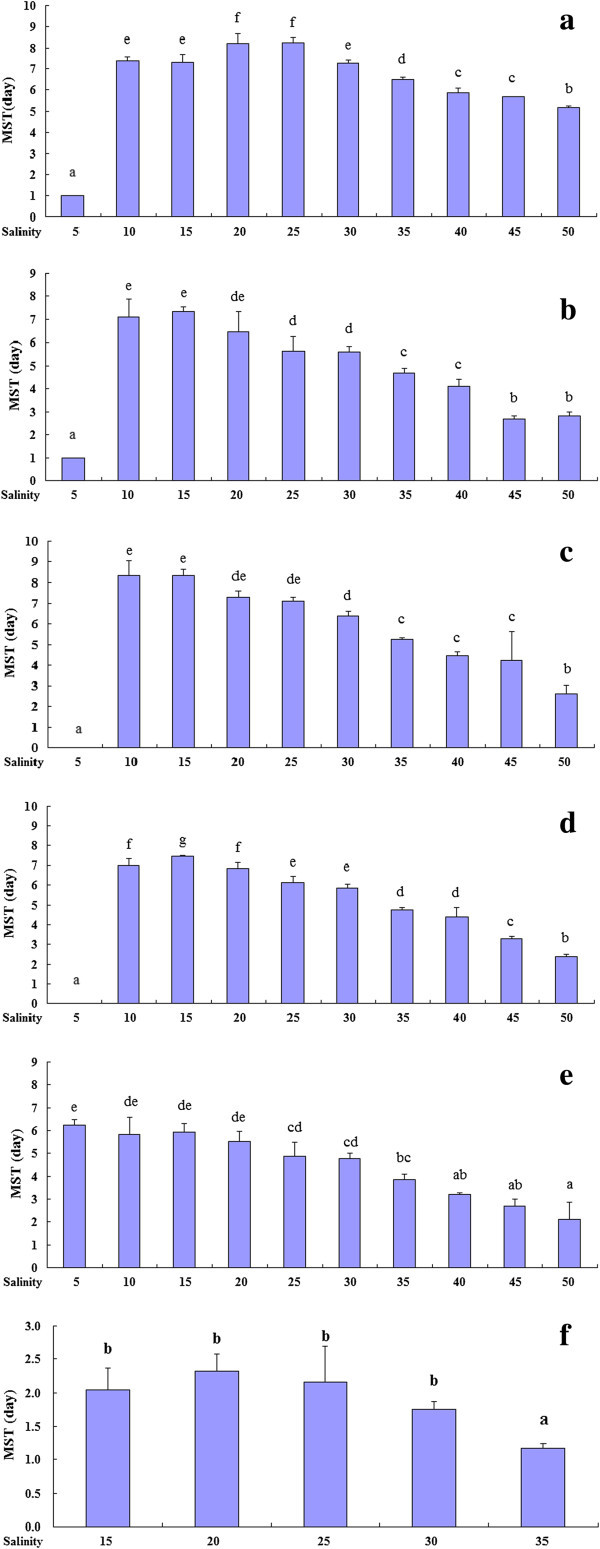
**Mean survival time under different salinities at different developmental stages in the*Inimicus japonicas,*a: newly hatched larva (1 DAH), b: yolk-sac larva (2 DAH), c: mouth-open larva (3 DAH), d: post yolk-sac larva (5 DAH), e: flexion larva (15 DAH) and f: juvenile (25 DAH).**

The MSTs of different developmental stages under various salinities were showed in Figure 6, and one-way ANOVA results were summarized in Table 2. For the newly hatched larvae, the MST under salinity 20‰ and 25‰ was significantly higher than that at other salinities, and was lowest at salinity 5‰ (Figure 6a). For the yolk-sac larvae, the lowest MST was under salinity 5‰, the highest MST was at salinity 15‰, and then decreased with salinity increase (Figure 6b). Similar trends were also found in mouth open and post-yolk sac larvae, both highest MST values were present at medium salinities (Figure 6c & d). However, flexion larvae showed high MST under salinity 5‰ to 20‰ (Figure 6e). In juvenile, the MST showed no significant difference among salinity 15–30, but lower at salinity 35‰ (Figure 6f).

## Discussion

Scorpaeniformes fish has two reproductive types, one is ovoviviparous, such as false kelpfish *Sebastiscus marmoratus*; and the other is fertilized externally, like *I. japonicus*. There are three types of teleost eggs, buoyant, sticky and demersal, and most marine teleostean spawn buoyant eggs. Usually one oil globule is contained in the egg, playing a role of floating. However, oil globule could not be found in *I. japonicus*, but the eggs were still floating in the seawater with a salinity of 30‰, indicating ups and downs of eggs of *I. japonicus* is related to the water content in the eggs. In this study, the full developmental sequence of the devil stinger *I. japonicus* from egg to juvenile in controlled aquarium conditions was stated. These results enabled us to compare the development and morphology of embryos of *I. japonicus* with those of other teleost fishes in detail. During the embryonic development of *I. japonicus*, the same events were observed as those seen in zebrafish *Danio rerio* (Kimmel et al. 1995), roughskin sculpin *Trachidermus fasciatus* (Takeshita et al. 1997; Wang et al. 2004), and cottid fish *Hemilepidotus gilberti* (Hayakawaa and Munehara 2001), and their stage definitions could be consistently adopted to describe the embryonic development of *I. japonicus*. Therefore, the embryonic development of *I. japonicus* can be considered to follow the general developmental pattern of teleosts. Egg size is an important consideration for egg and larval quality during incubation and rearing in aquaculture. The average diameter of most Scorpaeniformes fish eggs are around 1.2–2.0 mm, however, the size range is wide. Egg diameters of some Scorpaeniformes fish were reported as: 1.2–1.3 mm for *I. japonicus* (Kadomura et al. 2006; Kim et al. 2012), 1.3 mm for non-copulatory sculpin *Hemilepidotus gilberti* (Hayakawaa and Munehara 2001), 1.5–1.78 (1.98–2.21) mm for roughskin sculpin, *Trachidermus fasciatus* (Wang et al. 2004; Takeshita et al. 1997). The egg of devil stinger is spherical, floating and has approximately 1.40 mm average diameter, which is similar to its previous reports. The egg size and fecundity are determined by several factors, i.e., broodstock age, broodstock size, feed and water quality (Celik et al. 2012).

In most fish species the blastomeres are regular in size and shape (Hall 2008). In the devil stinger, first five cleavages divided the blastodisc into 32 equal-sized blastomeres at the animal pore and horizontal cleavage occurred between 64 and 128 cell stages (after the fifth division). In zebrafish *Danio rerio* (Kimmel et al. 1995), Atlantic cod *Gadus morhua* (Hall et al. 2004), and cichlid fish *Cichlasoma dimerusn* (Meijide and Guerrero 2000), the first horizontal cleavage occurs at the sixth cleavage, between the 32 and the 64 cell stages. It occurs between the 16 and 32 cell stages in the medaka *Oryzias latipes* (Iwamatsu 1994) and common snook *Centropomus undecimalis* (Yanes-Roca et al. 2012). It occurs even earlier in the Holostean fish *Amia calva* (between the 8 and the 16 cell stages) (Ballard 1986; Nakatsuji et al. 1997) and in the ice goby *Leucopsarion petersii* (between the 4 and the 8 cell stages) (Nakatsuji et al. 1997). Theoretical knowledge of embryonic development stages might be useful for incubation management with regard to environmental variables, thus larvae malformation and low productivity in captivity can be prevented (Celik et al. 2012). Furthermore, the information on embryonic and early larval development is important for large-scale seed production and aquaculture (Koumoundouros et al. 2001; Saillant et al. 2001). Teleost gastrulation was morphologically characterized by the presence of a germ ring (Arezo et al. 2005). In this study, gastrulation was observed at 11:36 hours and 50% epiboly began 13:45 h. *I. japonicus* embryo reached the eight-somite stage at 25:46 hours and reached the pre-hatching stage at 42 h with muscular contractions.

The development of teleost fins during incubation process is various among different species. For example, the fins of Salmonidae fish begin to develop before hatching, but the fins of other fish, such as *Nibea albiflora*, *Paralichthys olivaceus*, *Scomberomorus niphonius* and *Engraulis japonicus,* start to develop after hatching, and the pectoral fin rays form late (Kendall et al. 1984). In *I. japonicus*, pectoral fin buds developed early at the late embryonic stage, showing a fan-shape film with black spot after hatching. The pectoral fin was larger than the head 3 days after hatching, and three melanin spots spread on the edge of the fin films. The larvae were inactive but short periods of swimming were observed. They started swimming freely within 3–4 days. While many marine fish larvae had two kinds of energy reserves, yolk and oil globule (Bjelland and Berit 2006), devil stinger has only yolk sac. The yolk sac is depleted within 3–4 days and the larvae start to feed exogenously before complete absorption of the yolk sac. Mouth opening was on the third day. Primordium of tail fin appeared at 6 DAH, at the moment the pectoral fin had developed very large, and ten nicks formed on the edge of the fin rays, with fuscous melanin in each ray. After 20 days, bright gold yellow stripes appeared on the large fan-shape pectoral fin. In juveniles, the last two fin rays were separate from others. Possibly the development of pectoral fin in *I. japonicus* was corresponding with its functions. During larval stage, the fish were pelagic in the middle-upper waters, and fan-shape pectoral fin played a role in balance. When ten nicks showed up in the pectoral fin, they made the swimming of the larvae more accurate and flexible, guaranteeing their feeding successful. In the post-larvae, they changed the free-swimming to nestling on the bottom, because the large pectoral fin made their swimming slow. However, the powerful pectoral fins make the fish move quickly for a short distance intermittently, facilitating its successful feeding. In the juvenile stage, fish transferred to benthonic life style completely, and swam slowly on the bottom of the water supporting by the two separate pectoral fin rays. The development of pectoral fins in *I. japonicus* is useful for enhancing the active search and predation efficiency of food organisms, which is similar to the pectoral fins of yellow croaker *Larimichthys crocea* and river loach *Triplophysa bleekeri* (Li and Yan 2009; Wang et al. 2010).

Early larval development of *I. japonicus* was divided into four main periods: Yolk-sac larva: the presence of a yolk sac ventrally in the body, between hatching and 4 DAH. Yolk sac was absorbed and larvae swam actively 3–4 days after hatching, and the onset of exogenous feeding occurred 3 days later. Post yolk-sac larva: this period began at absorption of yolk sac and ended at the start of upward flexion of the notochord (between 4 and 12 DAH). Flexion larva: this period (the period during notochord flexion) was characterized with the hypural bones assuming a vertical position, between 13 and 15 DAH. Postflexion larva: the period between completion of flexion and the juvenile stage, 16–25 DAH. Our findings may provide a basis for further studying the complete early life history of *I. japonicus* and commercial production of this fish. The results of this study can contribute to a better understanding of the embryonic and larval development of other commercial scorpionfish larvae. They can be used to explain some aspects of the early life history at culture conditions and to develop better larval culture methodologies in hatchery. Similarly, they will be helpful to increase success rates in the larval culture of some scorpionfish fish species.

In the present study, based on salinity tolerance rest, salinity tolerance of *I. japonicus* was comparative wide, ranging from 10–30‰, the optimum salinity range was 10–20‰. Reducing the salinity appropriately did not negatively affect the development and growth of the larvae, but increased the survival of the larvae. This result was similar to the other fish species, such as *Nibea miichthioides* (Huang et al. 1997) and *Pagrosomus major* (Wang 2002). The SAI and MST are popular indexes for evaluating the vitality and quality of the larvae during the marine fish larviculture. In the present study, their values were higher at the salinities of 10–20‰, and were lower when salinity was below 10‰ or above 25‰. During the observation, larval development was normal under such salinity levels, indicating SAI and MST could be regarded as useful indicators for evaluating the optimum salinity range. Lin (2008) reported that suitable salinity range for *I. japonicus* larvae was 19–31‰, but he did not test the difference of salinity tolerance among different developmental stages, which was observed in the present study. The suitable salinity range for newly hatched larvae was 10–30‰. However, the suitable salinities for the yolk-sac larvae, mouth open larvae, and post yolk-sac larvae were almost the same, ranging from 10‰ to 20‰. The flexion larvae showed stronger low salinity tolerance compared with earlier stages, but this capacity decreased when the larvae finished metamorphosis. Except flexion larvae, all larvae were not able to survive at salinity 5‰, but lowering salinity appropriately could increase the survival of larvae in all developmental stages. Thus, in the present study, as a coastal fish species, the suitable salinity range for larviculture of *I. japonicus* was proved good at 10–20‰.

The SAI and MST displayed a similar trend under different salinities for all developmental stages. The SAI and MST are related to not only the nutrient storage, but also the living conditions. For example, when the salinity is suitable, the larvae only need to consume a little energy for osmoregulation, allocating large amount of energy to organ development and growth, thus survive longer under such conditions. However, when larvae are subject to lower or higher salinities, they need to spend more energy maintaining osmotic balance, and the other physiological functions are also affected, resulting in slow growth, reduced SAI and MST. In the present study, newly hatched larvae showed high SAI and MST at salinity 10–25‰, and larvae in other developmental stages showed higher values of the two parameters at salinity 10–20‰, indicating that the suitable salinity for the larviculture of *I. japonicus* should be reconsidered. Thus, the current salinity condition (30‰) in larviculture of Japanese devil stinger should be improved, and it is beneficial to reduce salinity moderately.
